# Development and Validation of Multicenter Predictive Nomograms for Locally Advanced Pancreatic Cancer After Chemoradiotherapy

**DOI:** 10.3389/fonc.2021.688576

**Published:** 2021-06-08

**Authors:** Xiaofei Zhu, Wenyu Liu, Yangsen Cao, Tingshi Su, Xixu Zhu, Yiyang Wang, Xiaoping Ju, Xianzhi Zhao, Lingong Jiang, Yusheng Ye, Huojun Zhang

**Affiliations:** ^1^ Department of Radiation Oncology, Changhai Hospital Affiliated to Navy Medical University, Shanghai, China; ^2^ Department of Hepatobiliary and Pancreatic Surgery, Changhai Hospital Affiliated to Naval Medical University, Shanghai, China; ^3^ Department of Radiation Oncology, Affiliated Tumor Hospital of Guangxi Medical University, Nanning, China; ^4^ Department of Radiation Oncology, General Hospital of Eastern Theater Command, Nanjing, China; ^5^ Department of Biostatistics, Shanghai Clinbrain Co. Ltd, Shanghai, China

**Keywords:** locally advanced pancreatic cancer, nomograms, chemoradiotherapy, overall survival, progression free survival

## Abstract

**Objective:**

Due to common practice of hypofractionated radiotherapy in pancreatic cancer and heterogeneous chemotherapy regimens in previous studies, modified nomograms are required. Therefore, we aim to develop and validate prognostic nomograms for locally advanced pancreatic cancer (LAPC) after stereotactic body radiation therapy (SBRT) and chemotherapy.

**Methods:**

The development cohort comprised 925 patients with LAPC receiving SBRT and gemcitabine-based chemotherapy in our center, while 297 patients from another two centers formed the validation cohort. Nomograms were created from COX models and internally validated by bootstrap. Model discriminations were evaluated by calibration plots and concordance index (C-index). A decision curve analysis (DCA) was performed to evaluate clinical benefits of nomograms. Additionally, recursive partitioning analysis (RPA) was used for stratifications of survival probability based on the total score of each patient calculated by nomograms.

**Results:**

Weight loss, tumor diameter, radiation dose, CA19-9 kinetics after treatment and surgical resection were included in the nomogram for overall survival (OS), while the five factors plus performance status formed the nomogram for progression free survival (PFS). The corrected C-indexes for estimated 1-year and 2-year OS of the development cohort were 0.88 (95% CI: 0.85-0.91) and 0.86 (95% CI: 0.83-0.90). For those of the validation cohort, it was 0.88 (95% CI: 0.82-0.94) and 0.83 (95% CI: 0.74-0.91). Additionally, the corrected C-index for predicted 1-year PFS in the development and validation cohort was 0.83 (95% CI: 0.81-0.86) and 0.82 (95% CI: 0.78-0.87), respectively. The calibration plots showed good agreement of 1- and 2-year OS and 1-year PFS between the estimations and actual observations. Potential clinical benefits were demonstrated with DCA. Additionally, for 1- and 2-year OS and 1-year PFS, patients were stratified into four groups with different survival probability by RPA.

**Conclusion:**

The validated nomograms provided useful predictions of OS and PFS for LAPC with chemoradiotherapy.

## Introduction

Pancreatic cancer still remains one of the most lethal malignancies and is fourth leading cause of cancer death in both genders in US, where the mortality and incidence increase over the past decade (1). Despite development of targeted therapies and immunotherapy, no significant survival benefits were found with a lowest 5-year survival rate of 9% among all cancers (1). Additionally, only about 20% patients with an initial diagnosis of early stage pancreatic cancer were candidates for upfront surgery. Regarding the rest patients with locally advanced pancreatic cancer (LAPC), chemoradiation may be the optimal treatment. Though it was demonstrated that no improved overall survival was found in chemoradiotherapy compared with chemotherapy alone in LAP07 (2), this still may be controversial due to a single agent as the chemotherapy regimen and conventional radiotherapy with relatively low single fraction dose and biological effective dose. Due to precise delivery of higher doses to tumors with stereotactic body radiation therapy (SBRT) and its shorter courses without delay of systemic treatment, SBRT has been commonly used in pancreatic cancer with favorable survival benefits. Therefore, it is pivotal to evaluate outcomes of patients receiving SBRT and standard chemotherapy, including FOLFIRINOX, gemcitabine plus nab-paclitaxel or S-1 which has been confirmed effective in Asians (3–6).

Additionally, patients in previous studies about prognosis prediction either underwent three dimensional conformal radiation therapy or intensity modulated radiotherapy or gemcitabine alone or other heterogeneous chemotherapy regimens (7, 8). Hence, a prognostic tool to accurately assess overall survival (OS) and progression free survival (PFS) of patients after the hypofractionated radiotherapy with uniform and standard chemotherapy regimen is required, which may provide a better management of patient care and improved quality of life. Therefore, the aim of our study was to develop and validate novel, multicenter predictive models based on a broad spectrum of factors.

## Methods

### Study Population

The development cohort consisted of consecutive patients with pathologically and radiographically proven LAPC receiving SBRT and chemotherapy, including gemcitabine plus nab-paclitaxel or S-1, in our center from January 2012 to December 2019 (**Supplementary Figure 1**). The definition of LAPC was referred to NCCN guideline: a) For tumor in the pancreatic head or uncinated process, solid tumor contact with the superior mesenteric artery >180° or celiac axis >180°, b) for tumor in the pancreatic body or tail, solid tumor contact of >180° with the superior mesenteric artery or celiac axis, or contact with celiac axis and aortic involvement, c) unreconstructible superior mesenteric venous or portal vein due to tumor involvement or occlusion. Furthermore, patients were eligible for inclusion only if pretreatment examinations or radiotherapy data were available and they were aged ≥ 18 years, and adequate bone marrow, hepatic, and renal function. Patients were excluded if they had a history of other malignancies or received prior radiotherapy or chemotherapy. The validation cohort was composed of patients with the same inclusion and exclusion criteria from other two centers.

Baseline characteristics were retrieved from the database prospectively maintained by the centers: age, gender, weight loss, smoking status, Eastern Cooperative Oncology Group (ECOG) performance status, tumor location, tumor diameter, CA19-9 levels, radiation doses, chemotherapy regimens and receiving surgery or not after chemoradiotherapy. The study was approved by the institutional review board of each study site and has therefore been performed in accordance with the ethical standards laid down in the 1964 Declaration of Helsinki and its later amendments. Informed consent was obtained from each patient before treatment.

### Data Definitions

The principal outcomes of interest were the predicted probability of 1-year and 2-year OS and 1-year PFS. OS and PFS were defined as time from the date of initiation of treatment to death from any cause and to local, regional, or distant progression or death from any cause, respectively. In addition to above baseline data, CA19-9 response and prognostic nutritional index (PNI) were also recorded. It was shown that PNI was predictive of prognosis of pancreatic cancer (9, 10). The formula of PNI was as follows: PNI = 10 × serum albumin (g/dl) + 0.005 × total lymphocyte count (/mm^3^).

The upper limit of normal for CA19-9 is usually considered as 37 U/ml (11). Moreover, it was demonstrated that decreases in CA19-9 levels of ≥50% correlated with an improved survival (12). Therefore, CA19-9 response was defined as a decrease of CA19-9 level by 50% from the baseline level of ≥74 U/ml. Patients were required to receive CA19-9 examinations every month during follow-up. The nadir value of CA19-9 level after treatment was utilized for the estimation of CA19-9 decrease. Hence, CA19-9 response was stratified as follows: CA19-9 levels ≥74 U/ml with response vs. CA19-9 levels ≥74 U/ml with no response (including CA19-9 levels within the normal range before treatment while increasing after treatment) vs. CA19-9 levels <74 U/ml all along before and after treatment, which have been used in our previous studies (13–15). Furthermore, it was reported that patients with a CA19-9 level less than 200U/ml had major response when they received neoadjuvant therapy (16). Therefore, the baseline level of CA19-9 was stratified as: <200U/ml and ≥200U/ml. Baseline C19-9 levels were determined one to three days before treatment initiations, while tumor diameters were measured within one week before the start of treatment. The interval between the diagnosis and start of treatment was one week. Besides, in our previous studies, it was demonstrated that BED_10_ (biological effective dose, α/β=10) ≥60Gy correlated with longer overall survival (OS) in patients with pancreatic cancer (13, 14, 17, 18). Therefore, BED_10_ was stratified into two groups for analysis: BED_10_ ≥60Gy and BED_10_ <60Gy.

### Treatment Delivery

Irradiation of pancreatic cancer was performed with SBRT *via* CyberKnife^®^ (Accuray Incorporated, Sunnyvale, CA) delivered by 5-8 fractions, as described previously (13–15, 18). Three gold fiducials within or adjacent to the pancreatic tumor were preferable. A radiographically evident gross disease was regarded as gross tumor volume (GTV). Clinical target volume (CTV) was defined as areas of the potential subclinical disease spread. In most cases, the CTV was equal to GTV. Planning target volume (PTV) included a 2-5mm margin on GTV or CTV. Additionally, if the tumor abutted to organs at risk, only 2mm margin expansions may be performed to reduce the risk of toxicities as possible. While 3-5mm margin expansions were allowed in the case of enough space between the tumor and organs at risk. Dose constraints of organs at risk were referred to the American Association of Physicists in Medicine guidelines in TG-101 (19). The initial contours at the time of SBRT were reviewed together by a radiation oncologist and a radiologist for accuracy. Triphasic CT was used to delineate tumor.

Chemotherapy was performed 2 to 3 weeks after SBRT. Patients were required to receive gemcitabine plus nab-paclitaxel or gemcitabine plus S-1. Gemcitabine (1000 mg/m^2^) and nab-paclitaxel (125 mg/m^2^) were administered on days 1, 8, and 15 during each 4-week cycle, which repeated for 4-6 cycles. S-1 was orally given at a dose of 80mg/m^2^ for 28 days followed by a 14-day rest, which also continued for 4-6 cycles.

### Statistical Analysis

Continuous and categorical data were expressed as median (range) and n (%), respectively. A student t-test or a Mann-Whitney U test was used for analysis in the case of normally or non-normally distributed continuous variables. Categorical variables were compared using the χ2 test or Fisher’s exact test. Factors with a P-value <0.05 in the univariate regression analysis were entered as candidate variables into multivariate COX regression analysis. Backward stepwise selection with the Akaike information criterion was used to identify potential variables for the multivariable logistic regression models. Those variables were incorporated in the nomogram. The final models were first internally validated by the bootstrap with 1000 resamples. The validation cohort from the other centers were used to externally validate the models. The accuracy of prediction of the models were evaluated with the calibration plots, which determined the agreement between the observed and estimated probability. The discrimination was assessed by the concordance index (C-index). The decision curve analysis was performed to evaluate clinical benefits of the nomograms compared with that of each risk factor. Additionally, recursive partitioning analysis was used for survival probability classifications of LAPC after chemoradiotherapy based on the total score of each patient calculated by the nomograms. A two-sided α of less than 0.05 was considered statistically significant. All analyses were performed using SPSS 22.0 (IBM Corporation, Armonk, NY) and the Regression Modeling Strategies package, version 5.1-4 in R, version 3.6.3.

## Results

### Characteristics of Development and Validation Cohort

A total of 925 and 297 patients were included in the development and validation cohort. The baseline characteristics of the two cohorts were summarized in **Table 1**. There were no differences in all patient characteristics between two cohorts. The median follow-up was 17.8 months (range: 4.1-47.9 months) and 19.3 months (range: 5.2-37.1 months), respectively. The median OS of development and validation cohort was 16.6 months (95% CI: 16.3-16.9 months) and 16.9 months (95% CI: 16.4-17.4 months), respectively. The 1-year OS of development and validation cohort was 88.1% (95% CI: 87%-89.2%) and 90.5% (95% CI: 88.8%-92.2%), respectively. The 2-year OS was 8.1% (95% CI: 7.2%-9.0%) and 7.1% (95% CI: 5.6%-8.6%), respectively. The median PFS was 11.3 months (95% CI: 11.0-11.6 months) and 11.4 months (95% CI: 11.0-11.8 months), respectively. The 1-year PFS was 42.4% (95% CI: 40.8%-44.0%) and 44.1% (95% CI: 41.2%-47.0%), respectively.

**Table 1:** Baseline characteristics of development and validation cohort.

Characteristics	Development cohort (n=925) No. (%)	Validation cohort (n=297) No. (%)	P value
Sex			0.951
Female	375 (40.5)	121 (40.7)	
Male	550 (59.5)	176 (59.3)	
Age, years			
Median	65	66	0.780
Range	26-90	35-88	
<65	414 (44.8)	129 (43.4)	0.690
≥65	511 (55.2)	168 (56.6)	
Weight loss, kg			0.891
No weight loss	223 (24.1)	67 (22.6)	
<5	262 (28.3)	87 (29.3)	
≥5	440 (47.6)	143 (48.1)	
Smoking			0.840
Non-smoker	638 (69.0)	203 (68.3)	
Smoker	287 (31.0)	94 (31.7)	
ECOG			
0-1 point	218 (23.6)	78 (26.3)	0.346
2-3 points	707 (76.4)	219 (73.7)	
Tumor location			
Head	660 (71.4)	213 (71.7)	0.903
Body or tail	265 (28.6)	84 (28.3)	
Tumor diameter, cm			
Median	3.6	3.6	0.508
Range	1.3-9.0	1.4-7.8	
<4	590 (63.8)	193 (65.0)	0.708
≥4	335 (36.2)	104 (35.0)	
CA19-9 level, U/ml			0.765
<200	430 (46.5)	146 (49.2)	
≥200	495 (53.5)	151 (50.8)	
PNI			0.878
<48	478 (51.7)	155 (52.2)	
≥48	447 (48.3)	142 (47.8)	
BED_10_, Gy			0.243
<60	448 (48.4)	132 (44.4)	
≥60	479 (51.8)	165 (55.6)	
Chemotherapy regimen			0.437
Gemcitabine + nab-paclitaxel	465 (50.3)	157 (52.9)	
Gemcitabine + S-1	460 (49.7)	140 (47.1)	
CA19-9 response			0.187
CA19-9 levels ≥74 U/ml with response	451 (48.7)	154 (51.9)	
CA19-9 levels <74U/ml all along	242 (26.2)	82 (27.6)	
CA19-9 levels ≥74 U/ml with no response	232 (25.1)	61 (20.5)	
Surgical resection			0.778
Yes	86 (9.3)	26 (8.8)	
No	839 (90.7)	271 (91.2)	

### Factors Predictive of OS and PFS

Regarding OS, weight loss, ECOG performance status, tumor diameter, BED_10_, CA19-9 response and surgical resection correlated with OS in the univariate analysis. Thereafter, backward stepwise selection in the multivariate analysis showed that only weight loss (No weight loss as reference; <5kg, HR: 1.23 [95% CI: 1.02-1.48], ≥5kg, HR: 1.42 [95% CI: 1.19-1.69], P<0.001), tumor diameter (<4cm as reference; ≥4cm, HR: 1.36 [95% CI: 1.18-1.56], P<0.001), BED_10_ (<60Gy as reference; ≥60Gy, HR: 0.35 [95% CI: 0.30-0.41], P<0.001), CA19-9 response (≥74 U/ml with response as reference; <74U/ml all along, HR: 1.05 [95% CI: 0.89-1.24], ≥74 U/ml with no response, HR: 3.84 [95% CI: 3.21-4.59], P<0.001) and surgical resection (No as reference; yes, HR: 0.38 [95% CI: 0.30-0.49], P<0.001) were predictive of OS (**Supplementary Table 1**).

Similarly, after multivariate analysis, it was clarified that weight loss (No weight loss as reference; <5kg, HR: 1.13 [95% CI: 0.94-1.36], ≥5kg, HR: 1.26 [95% CI: 1.07-1.50], P=0.024), ECOG performance status (0-1 point as reference; 2-3 points, HR: 1.18 [95% CI: 1.01-1.39], P=0.036), tumor diameter (<4cm as reference; ≥4cm, HR: 1.32 [95% CI: 1.15-1.51], P<0.001), BED_10_ (<60Gy as reference; ≥60Gy, HR: 0.37 [95% CI: 0.32-0.43], P<0.001), CA19-9 response (≥74 U/ml with response as reference; <74U/ml all along, HR: 0.91 [95% CI: 0.77-1.07], ≥74 U/ml with no response, HR: 3.22 [95% CI: 2.71-3.84], P<0.001) and surgical resection (No as reference; yes, HR: 0.43 [95% CI: 0.34-0.55], P<0.001) were predictive of PFS (**Supplementary Table 2**).

### Performance of the Nomogram

**Figure 1:**
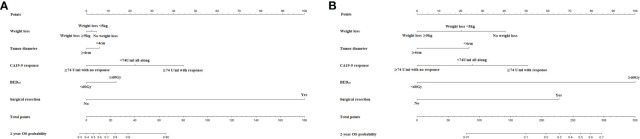
**(A)** The nomogram for predicting probability of OS at 1 year and **(B)** 2 years.

**Figure 2:**
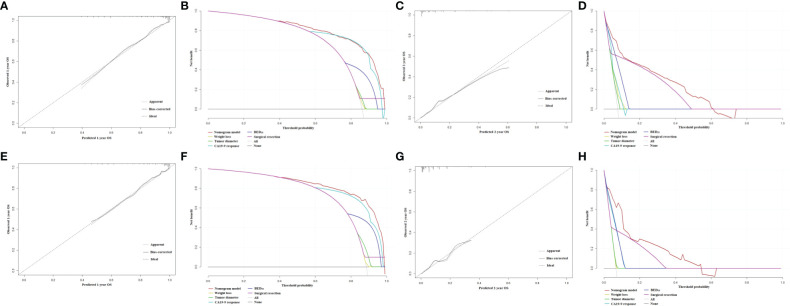
**(A)** The calibration plot of OS at 1 year in the development cohort. **(B)** Net benefit in relation to threshold probability of OS at 1 year in the development cohort. **(C)** The calibration plot of OS at 2 years in the development cohort. **(D)** Net benefit in relation to threshold probability of OS at 2 years in the development cohort. **(E)** The calibration plot of OS at 1 year in the validation cohort. **(F)** Net benefit in relation to threshold probability of OS at 1 year in the validation cohort. **(G)** The calibration plot of OS at 2 years in the validation cohort. **(H)** Net benefit in relation to threshold probability of OS at 2 years in the validation cohort.

The nomograms for prediction of 1-year and 2-year OS were shown in **Figure 1**. The calibration plots demonstrated good agreement of 1-year and 2-year OS between the estimations of nomograms and actual observations in the development cohort (**Figures 2A–C**). Regarding discriminative ability, the bootstrap corrected C-index of nomograms for estimated 1-year and 2-year OS in the development cohort was 0.88 (95% CI: 0.85-0.91) and 0.86 (95% CI: 0.83-0.90), respectively. The decision curves showed satisfactory positive net benefits of the two nomograms at the threshold probability compared with other factors (**Figures 2B–D**). For external validation, the calibration curves also demonstrated favorable consistency between the predicted and observed probability (**Figures 2E–G**). The corrected C-index of nomograms for predicted 1-year and 2-year OS in the validation cohort was 0.88 (95% CI: 0.82-0.94) and 0.83 (95% CI: 0.74-0.91), respectively. Similarly, potential clinical benefits of the two nomograms were also demonstrated with the decision curves (**Figures 2F–H**).

**Figure 3:**
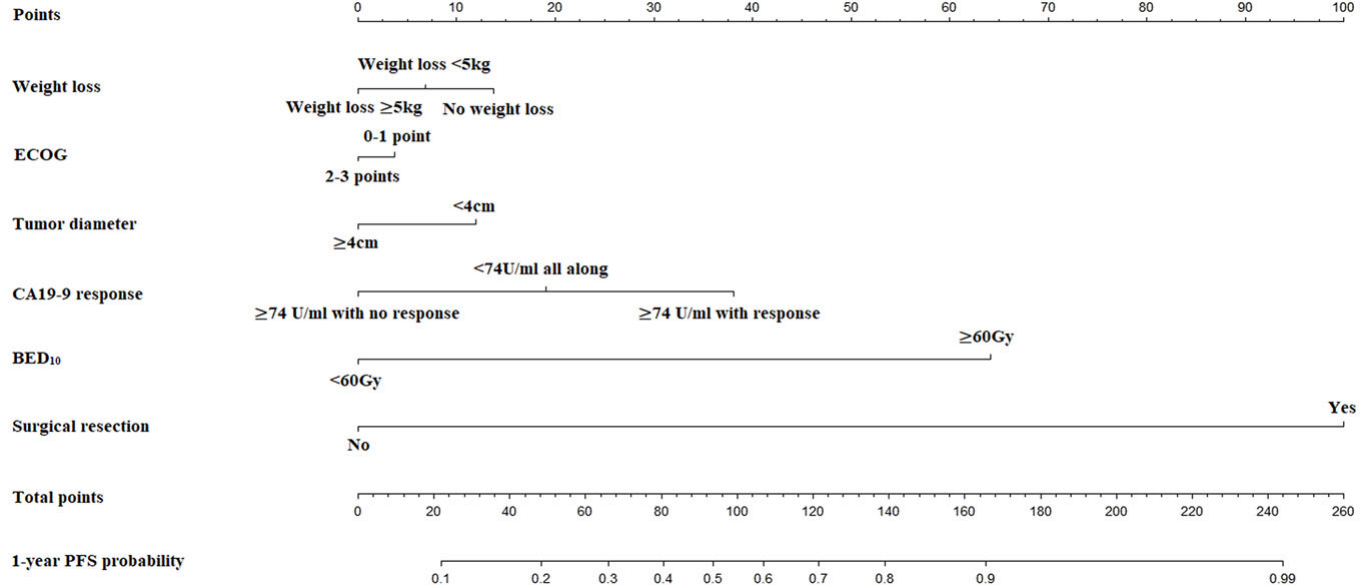
The nomogram for predicting probability of PFS at 1 year.

**Figure 4:**
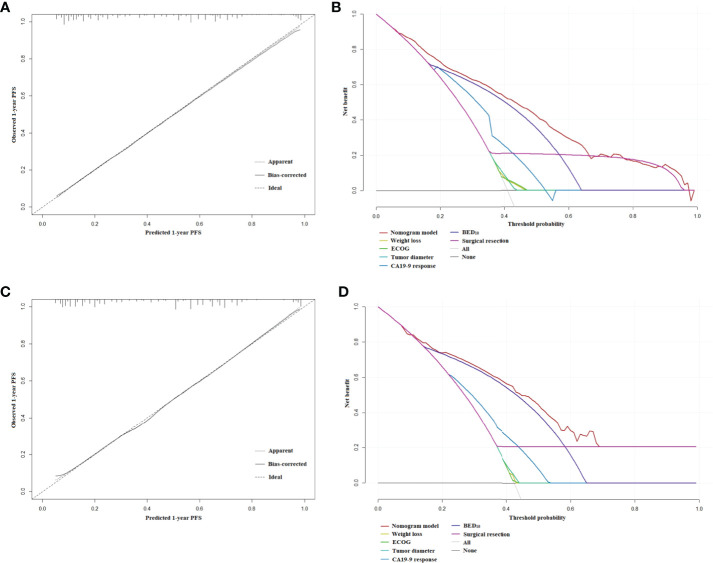
**(A)** The calibration plot of PFS at 1 year in the development cohort. **(B)** Net benefit in relation to threshold probability of PFS at 1 year in the development cohort. **(C)** The calibration plot of PFS at 1 year in the validation cohort. **(D)** Net benefit in relation to threshold probability of PFS at 1 year in the validation cohort.

The nomogram for estimated 1-year PFS was illustrated in **Figure 3**. The prediction agreed well with the observation in the calibration plots of development and validation cohort (**Figures 4A–C**). Additionally, the corrected C-index of the development and validation cohort was 0.83 (95% CI: 0.81-0.86) and 0.82 (95% CI: 0.78-0.87), respectively. Furthermore, significant net benefits were found in the two cohorts (**Figures 4B–D**).

### Stratifications of Probability of OS and PFS Based on Nomograms

**Figure 5:**
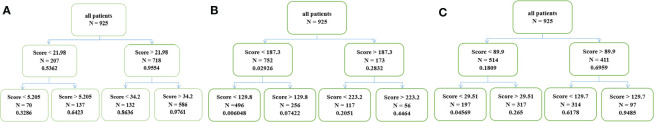
**(A)** Four groups with different survival probability of OS at 1 year, **(B)** 2 years and **(C)** PFS at 1 year.

Novel classifications of estimated 1-year and 2-year OS and 1-year PFS were shown in **Figure 5**. All patients were stratified into four groups based on total scores calculated by nomograms, which were low, low-intermediate, intermediate-high and high probability of 1- and 2-year OS and 1-year PFS groups. Cut-off scores and probabilities were also illustrated in **Figure 5**.

## Discussion

In this study, we develop and validate nomograms predicting 1- and 2-year OS and 1-year PFS for patients with LAPC receiving SBRT and chemotherapy. Notably, this pilot study focused on a major population of pancreatic cancer and evaluated outcomes of an intensified treatment modality with hypofractionated radiotherapy and standard chemotherapy. Additionally, the nomograms were beneficial for patient-specific estimated of OS and PFS that can be used for stratifications of survival probability and assessment of prognosis for patients.

Although previous studies have investigated nomograms for advanced pancreatic cancer (7, 8, 20, 21), chemotherapy regimens in most studies were heterogeneous, which may influence outcomes (7, 20, 21). Furthermore, patients in those studies underwent chemotherapy alone other than combination therapy, which may provide better local control and more survival benefits (20, 21). Besides, no external validations were performed in two nomograms (7, 21). Additionally, Vernerey et al. included patients of LAP07 as the development cohort, where patients either received chemoradiotherapy or chemotherapy (8). Moreover, three-dimensional conformal radiation therapy was applied in this study, which was not a mainstay modality of radiotherapy in the case of intensified modulated radiotherapy or SBRT commonly used in LAPC (8). Most importantly, SBRT has already been recommended in the management of pancreatic cancer according to American Society for Radiation Oncology clinical practice guideline for pancreatic cancer (22). Hence, evaluations of patients’ outcomes after standard chemotherapy regimens with the novel radiotherapy technique are required.

Compared with those published nomograms, our internally and externally validated nomograms have some similarities and potential advantages. Several predictive factors in our nomogram were common with them, including performance status, tumor size, radiation dose and surgical resection. However, our larger sample size of LAPC, longer follow-up and prospective data collection allow us to investigate contributions of other factors to survival and develop separate nomograms for 1- and 2-year OS and 1-PFS, while most of the studies only predicted short-term OS (7, 8, 20, 21). In addition, clinical net benefits were estimated by decision curve analysis and patients were classified into four groups of different OS and PFS probability with recursive partitioning analysis, which may be in favor of accurate personalized evaluations of prognosis and decision making of treatment. Unfortunately, this had not been discussed in previous studies.

It was demonstrated that CA19-9 level correlated with survival of patients with pancreatic cancer, which was confirmed in published nomograms where baseline CA19-9 level was included as a predictor (7, 8, 20). However, dynamic changes of CA19-9 level after treatment are usually considered as the surveillance of pancreatic cancer. A significant decrease may indicate effective treatment while a dramatic increase may imply disease progressions, which may be more sensitive than imaging examinations. Therefore, CA19-9 response was included in our nomogram. It was clarified that significant decrease of CA19-9 levels after treatment was predictive of better outcomes. As a result, it suggested that patients could still achieve survival benefits despite high pre-treatment CA19-9 levels if they responded well to treatment. This may result in a discrepancy between previous and our nomograms. For the former ones, patients with elevated baseline CA19-9 levels were all stratified into inferior survival groups, which may reduce the predictive accuracy of prognostication for an individual patient.

Furthermore, we identified that some of patients in our study become candidates for surgical resection after chemoradiotherapy, which could lead to longer OS. This was only proven in a previous study, where chemoradiotherapy was delivered (7). However, for the rest studies with systemic therapy alone, surgical resection was not performed and included in the nomogram (8, 20, 21). In a previous study with review of The National Cancer Data Base, it was demonstrated that the addition of radiotherapy to neoadjuvant chemotherapy was associated with higher downstaging and complete pathologic response rates than chemotherapy alone (23). Additionally, there was about 6.0-14.4% downstaging rate after chemoradiotherapy for LAPC (23–25), which was similar to that (9.3%) in our study. Nevertheless, chemoradiation did not confer a survival benefit compared with chemotherapy alone (23). This may be ascribed to conventional fractionation of radiotherapy and relatively low dose. Hence, chemoradiation should be considered as multimodality treatment for LAPC but requires further investigations.

In our nomogram, a high dose may provide favorable OS and PFS, which was proved in previous studies (14, 26, 27). Similarly, the radiation dose was also a predictor in the nomogram in a published one (7). The median OS and 2-year OS rates of patients with BED_10_ >70Gy in our center (data not published) were consistent with the ones in Krishnan et al. (27) (median OS: 20.3 months vs. 17.8 months, 2-year OS rate: 36.8% vs. 36.0%, 3-year OS rate: 18.7% vs. 31.0%). However, there were controversial results from the meta-analysis (28). The median OS of two included studies with the most weight employing BED_10_ >70Gy was 12.5 months and 10.3 months, while the median OS of another two studies with the most weight where a BED_10_ of 60-70Gy was used was 13.9 months and 15.0 months, respectively. The controversial result could be attributed to the different radiosurgery platforms and chemotherapy regimens. Hence, the interpretations of the results may not negatively impact clinical practice with dose escalation as a therapeutic paradigm for LAPC.

We acknowledged several limitations. First, patients in our study did not receive FOLFIRINOX, which may be given the first priority in chemotherapy regimens. However, patients receiving FOLFIRINOX may experience more severe adverse effects than gemcitabine-based chemotherapy. Furthermore, S-1 is not recommended in the guideline but it has been proven effective in Asians (3–6). Therefore, the nomogram could not be used for patients with FOLFIRINOX. Second, molecular profiles were not available in the study and could not be investigated as predictors of outcomes. Nonetheless, our recent study showed that high signal intensity of Ki-67, P53 and PD-L1 were associated with worse prognosis (17). Hence, these signatures would be assessed and included in our next-generation nomogram. Third, due to no optimal treatment for LAPC, SBRT as a radiation technique may result in limited clinical practice of nomograms owing to that conventional radiotherapy was still used in some centers. Moreover, we cannot exclude all possibility of residual confounding after internal validation as a result of possible overfitting from variable and threshold selection for these models. However, internal validation with bootstrapping and external validation could address the concerns. Due to nomograms that were developed based on information after treatment, nomograms could be used for prediction of patients’ outcomes when they were deemed to be candidates for SBRT plus chemotherapy, other than treatment recommendation.

In the context of rapid evolving radiotherapy approaches and more concerns about chemoradiation for LAPC, our nomograms are able to predict outcomes of LAPC and indicate accurately potential clinical benefits patients achieve after chemoradiotherapy. They may be helpful for physicians during treatment decision-making and highly tailored patient management in clinical practice.

## Data Availability Statement

The raw data supporting the conclusions of this article will be made available by the authors, without undue reservation.

## Author Contributions

HZ designed the study. XFZ and WL drafted the manuscript. YC optimized the radiation plans. TS, XXZ, XZZ, LJ, and YY performed patients’ follow-up. XFZ and YW performed the statistical analyses and interpreted data. XJ revised the manuscript. All authors contributed to the article and approved the submitted version.

## Funding

The study was funded by Shanghai Shenkang Center Innovation Research Program (SHDC2020CR3087B) and Changhai Hospital Clinical Investigation Program (2019YPT004).

## Conflict of Interest

YW was employed Shanghai Clinbrain Co. Ltd.

The remaining authors declare that the research was conducted in the absence of any commercial or financial relationships that could be construed as a potential conflict of interest.

## Supplementary Material

Supplementary Figure 1 Flow diagram.
