# Supplementary figures and images for: Development and Validation of Multicenter Predictive Nomograms for Locally Advanced Pancreatic Cancer After Chemoradiotherapy
Source: Front Oncol. 2021 Jun 8;11:688576. doi: 10.3389/fonc.2021.688576 (PMC8217648; doi:10.3389/fonc.2021.688576)

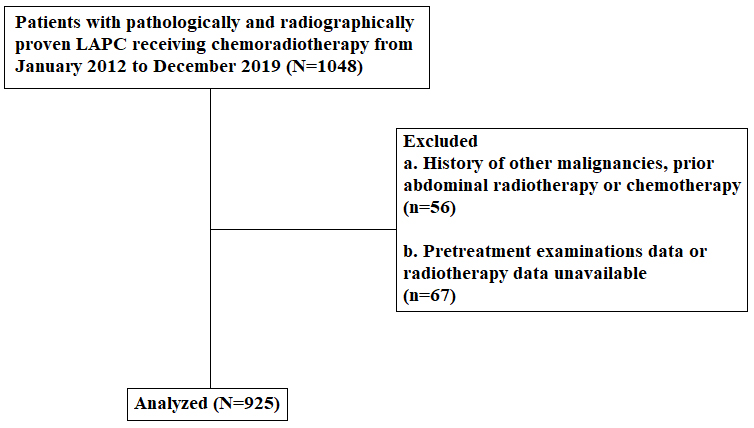

Supplement: Supplementary Figure 1 — Flow diagram. [file Image_1.tif]
