# Supplementary material for: Development and Validation of Multicenter Predictive Nomograms for Locally Advanced Pancreatic Cancer After Chemoradiotherapy
Source: Front Oncol. 2021 Jun 8;11:688576. doi: 10.3389/fonc.2021.688576 (PMC8217648; doi:10.3389/fonc.2021.688576)
Supplement: Supplementary file 2 [file Table_1.docx]

Table 1. Factors predicting OS

| Factors | Univariate analysis | | Multivariate analysis | |
| --- | --- | --- | --- | --- |
|  | HR (95% CI) | P value | HR (95% CI) | P value |
| Sex |  | 0.301 |  |  |
| Female | 1 (ref) |  |  |  |
| Male | 0.93 (0.81-1.07) |  |  |  |
| Age, years |  | 0.363 |  |  |
| <65 | 1 (ref) |  |  |  |
| ≥65 | 1.06 (0.93-1.22) |  |  |  |
| Weight loss, kg |  | 0.001 |  | <0.001 |
| No weight loss | 1 (ref) |  | 1 (ref) |  |
| <5 | 1.20 (0.99-1.44) |  | 1.23 (1.02-1.48) |  |
| ≥5 | 1.37 (1.15-1.62) |  | 1.42 (1.19-1.69) |  |
| Smoking |  | 0.299 |  |  |
| Non-smoker | 1 (ref) |  |  |  |
| Smoker | 0.93 (0.80-1.07) |  |  |  |
| ECOG |  | 0.011 |  |  |
| 0-1 point | 1 (ref) |  |  |  |
| 2-3 points | 1.22 (1.05-1.43) |  |  |  |
| Tumor diameter, cm |  | 0.008 |  | <0.001 |
| <4 | 1 (ref) |  | 1 (ref) |  |
| ≥4 | 1.21 (1.05-1.38) |  | 1.36 (1.18-1.56) |  |
| CA19-9 level, U/ml |  | 0.312 |  |  |
| <200 | 1 (ref) |  |  |  |
| ≥200 | 1.07 (0.94-1.22) |  |  |  |
| PNI |  | 0.081 |  |  |
| <48 | 1 (ref) |  |  |  |
| ≥48 | 0.89 (0.78-1.01) |  |  |  |
| BED_10_, Gy |  | <0.001 |  | <0.001 |
| <60 | 1 (ref) |  | 1 (ref) |  |
| ≥60 | 0.32 (0.28-0.37) |  | 0.35 (0.30-0.41) |  |
| Chemotherapy regimen |  | 0.746 |  |  |
| Gemcitabine + nab-paclitaxel | 1 (ref) |  |  |  |
| Gemcitabine + S-1 | 0.98 (0.86-1.12) |  |  |  |
| CA19-9 response |  | <0.001 |  | <0.001 |
| CA19-9 levels ≥74 U/ml with response | 1 (ref) |  | 1 (ref) |  |
| CA19-9 levels <74U/ml all along | 1.10 (0.94-1.30) |  | 1.05 (0.89-1.24) |  |
| CA19-9 levels ≥74 U/ml with no response | 4.82 (4.05-5.74) |  | 3.84 (3.21-4.59) |  |
| Surgical resection |  | <0.001 |  | <0.001 |
| No | 1 (ref) |  | 1 (ref) |  |
| Yes | 0.31 (0.25-0.39) |  | 0.38 (0.30-0.49) |  |

Table 2. Factors predicting PFS

| Factors | Univariate analysis | | Multivariate analysis | |
| --- | --- | --- | --- | --- |
|  | HR (95% CI) | P value | HR (95% CI) | P value |
| Sex |  | 0.725 |  |  |
| Female | 1 (ref) |  |  |  |
| Male | 0.98 (0.86-1.11) |  |  |  |
| Age, years |  | 0.824 |  |  |
| <65 | 1 (ref) |  |  |  |
| ≥65 | 1.02 (0.89-1.16) |  |  |  |
| Weight loss, kg |  | 0.007 |  | 0.024 |
| No weight loss | 1 (ref) |  | 1 (ref) |  |
| <5 | 1.17 (0.97-1.40) |  | 1.13 (0.94-1.36) |  |
| ≥5 | 1.31 (1.11-1.54) |  | 1.26 (1.07-1.50) |  |
| Smoking |  | 0.379 |  |  |
| Non-smoker | 1 (ref) |  |  |  |
| Smoker | 0.94 (0.81-1.08) |  |  |  |
| ECOG |  | 0.001 |  | 0.036 |
| 0-1 point | 1 (ref) |  | 1 (ref) |  |
| 2-3 points | 1.29 (1.10-1.50) |  | 1.18 (1.01-1.39) |  |
| Tumor diameter, cm |  | 0.003 |  | <0.001 |
| <4 | 1 (ref) |  | 1 (ref) |  |
| ≥4 | 1.23 (1.07-1.41) |  | 1.32 (1.15-1.51) |  |
| CA19-9 level, U/ml |  | 0.085 |  |  |
| <200 | 1 (ref) |  |  |  |
| ≥200 | 1.12 (0.98-1.28) |  |  |  |
| PNI |  | 0.093 |  |  |
| <48 | 1 (ref) |  |  |  |
| ≥48 | 0.89 (0.78-1.02) |  |  |  |
| BED_10_, Gy |  | <0.001 |  | <0.001 |
| <60 | 1 (ref) |  | 1 (ref) |  |
| ≥60 | 0.34 (0.30-0.39) |  | 0.37 (0.32-0.43) |  |
| Chemotherapy regimen |  | 0.880 |  |  |
| Gemcitabine + nab-paclitaxel | 1 (ref) |  |  |  |
| Gemcitabine + S-1 | 0.99 (0.87-1.13) |  |  |  |
| CA19-9 response |  | <0.001 |  | <0.001 |
| CA19-9 levels ≥74 U/ml with response | 1 (ref) |  | 1 (ref) |  |
| CA19-9 levels <74U/ml all along | 0.99 (0.84-1.16) |  | 0.91 (0.77-1.07) |  |
| CA19-9 levels ≥74 U/ml with no response | 4.01 (3.38-4.76) |  | 3.22 (2.71-3.84) |  |
| Surgical resection |  | <0.001 |  | <0.001 |
| No | 1 (ref) |  | 1 (ref) |  |
| Yes | 0.35 (0.28-0.44) |  | 0.43 (0.34-0.55) |  |
